# *KRAS *rs61764370 is associated with HER2-overexpressed and poorly-differentiated breast cancer in hormone replacement therapy users: a case control study

**DOI:** 10.1186/1471-2407-12-105

**Published:** 2012-03-22

**Authors:** Jasmina-Ziva Cerne, Vida Stegel, Ksenija Gersak, Srdjan Novakovic

**Affiliations:** 1 Institute of Medical Genetics, Department of Obstetrics and Gynecology, University Medical Center Ljubljana, Slajmerjeva 3, Ljubljana 1000, Slovenia; 2 Department of Molecular Diagnostics, Institute of Oncology Ljubljana, Zaloska 2, Ljubljana 1000, Slovenia

**Keywords:** *KRAS *rs61764370, Breast cancer, Tumor characteristics, Hormone replacement therapy

## Abstract

**Background:**

A single nucleotide polymorphism located in the 3'-untranslated region of the *KRAS *oncogene (*KRAS *variant; rs61764370) disrupts a let-7 miRNA binding and was recently reported to act as a genetic marker for increased risk of developing human cancers. We aimed to investigate an association of the *KRAS *variant with sporadic and familial breast cancer and breast tumor characteristics.

**Methods:**

Genotyping was accomplished in 530 sporadic postmenopausal breast cancer cases, 165 familial breast cancer cases (including N = 29, who test positive for *BRCA1/2 *mutations) and 270 postmenopausal control women using the flurogenic 5' nuclease assay. Information on hormone replacement therapy (HRT) use and tumor characteristics in sporadic breast cancer cases was ascertained from a postal questionnaire and pathology reports, respectively. Associations between the *KRAS *genotype and breast cancer or breast tumor characteristics were assessed using chi-square test and logistic regression models.

**Results:**

No evidence of association was observed between the *KRAS *variant and risk of sporadic and familial breast cancer - either among *BRCA *carriers or non-*BRCA *carriers. The *KRAS *variant was statistically significantly more often associated with human epidermal growth factor receptor 2 (HER2) - positive tumors and tumors of higher histopathologic grade. However, both associations were detected only in HRT users.

**Conclusion:**

Our data do not support the hypothesis that the *KRAS *variant rs61764370 is implicated in the aetiology of sporadic or of familial breast cancer. In postmenopausal women using HRT, the *KRAS *variant might lead to HER2 overexpressed and poorly-differentiated breast tumors, both indicators of a worse prognosis.

## Background

MicroRNAs (miRNAs) are a class of small non-coding RNAs that function as negative gene regulators. Depending on the degree of complementarity between the miRNA and its target mRNA, miRNAs post-transcriptionally regulate target gene expression by either inhibiting mRNA translation or inducing mRNA degradation [1]. Recent evidence has shown that impaired miRNA expression or single nucleotide polymorphisms (SNPs) that reside on miRNAs and/or miRNA-binding sites correlate with various human cancers [2]. Depending on target mRNAs, miRNAs can function as tumor suppressors or oncogenes [3].

The let-7 family of miRNAs plays an important role in tumorigenesis by regulating the expression of multiple oncogenes, including *KRAS *[4]. A germline SNP rs61764370 is located in the 3'-untranslated region of the *KRAS *oncogene and is referred to as the *KRAS *variant. The *KRAS *variant was demonstrated to be functional by disrupting a let-7 miRNA-binding site, and therefore leading to increased KRAS levels *in vitro *[5]. The same group also identified the *KRAS *variant to be associated with 2.3-fold increased risk for non-small-cell lung cancer (NSCLC) among moderate smokers [5]. By other report, tumors containing the *KRAS *variant allele had lower let-7 levels, which has been associated with shortened postoperative survival in NSCLC [6]. The presence of the *KRAS *variant was likewise associated with poor prognosis in head and neck squamous cell carcinoma as well as with the 2.5-fold increased risk of developing epithelial ovarian cancer (EOC) [7,8]. The variant allele was identified in 25% of non-selected EOC cases and in 61% of EOC patients from hereditary breast and ovarian cancer (HBOC) families previously considered uninformative for gene mutations [8]. However, data from a subsequent meta-analysis excluded the possibility of an association between the *KRA*S variant and a clinically significant risk of unselected, serous, familial EOC, or EOC among women carrying deleterious mutations in *BRCA1 *[9].

Since the *KRAS *variant was reported to be enriched in ovarian cancer patients from HBOC families, the study by Hollestelle and colleagues further investigated the frequency of the *KRAS *variant in breast cancer families [10]. As compared to the presence of the variant allele among controls (17.3%), the increased frequency of the *KRAS *variant was confirmed in breast cancer cases from *BRCA1 *families (23.5%), but not among breast cancer cases from *BRCA2 *(13.5%) or non-*BRCA1/2 *families (15.8%) [10]. Another study found the *KRAS *variant to act as a genetic marker for increased risk of developing triple negative breast cancer in premenopausal women (OR 2.31, 95% CI 1.26-4.22) [11].

On the basis of the current evidence, the purpose of our study was to investigate the association of the *KRAS *variant with sporadic and familial breast cancer risk among Slovenian women. Furthermore, we aimed to investigate the association of the *KRAS *variant with breast tumor characteristics among Slovenian postmenopausal sporadic breast cancer cases stratified by hormone replacement therapy (HRT) use.

## Patients and methods

### Study population

Participants were those of our previous breast cancer case-control study [12]. In brief, we enrolled postmenopausal women, who were 50-69 years old and of Caucasian ethnic origin. Cases diagnosed with invasive primary breast cancer were enrolled from the Institute of Oncology Ljubljana. Control women were randomly recruited from the outpatient clinic records of the Department of Obstetrics and Gynecology, University Medical Centre Ljubljana during their routine gynecologic exams. Response rates and exclusion criteria for the participants have been published previously [12].

The present analysis includes also a cohort of familial breast cancer cases, who underwent genetic testing between 2009-2011 at the Institute of Oncology Ljubljana.

Informed written consent was obtained from all women enrolled in the study. The study protocol was approved by the National Medical Ethics Committee of the Republic of Slovenia (No. 61/06/07).

### Data collection

Women enrolled in breast cancer case-control study were invited to participate through a postal questionnaire. Detailed questions were asked regarding sex hormone intake, with an emphasis on HRT use. A color chart displaying all preparations ever marketed in Slovenia was included in the questionnaire to aid recall. Information was obtained on the duration of HRT use (3 categories: less than 1 year, short-term use: 1 < 5 years, long-term use: 5 or more years) and regimen of HRT use (estrogen therapy, estrogen plus progestin therapy - there was no tibolone or other non-estrogen user). Users of systemic and/or local route of HRT administration were included in the analyses. HRT use for less than 1 year was considered no use. Women were considered postmenopausal if they had self-reported their last menstrual bleeding being at least 12 months before the reference date or had undergone a bilateral oophorectomy.

Information on tumor characteristics in sporadic breast cancer cases was retrieved from pathology reports in the patient's medical records. Tumor grading was performed according to the Nottingham scheme [13]. This grading method evaluates three parameters and assigns a score of 1 to 3 for each parameter: tubule formation (> 75% = 1, 10% to 75% = 2, < 10% = 3), nuclear atypia (none = 1, moderate = 2, marked = 3), and number of mitoses per 10 high-power fields (HPF), based on a HPF size of 0.274 mm^2 ^(< 10 mitoses = 1, 10 to 19 mitoses = 2, ≥ 20 mitoses = 3). The final Nottingham histologic grade is based on the sum of scores of the three parameters: 3, 4 or 5 = grade 1, 6 or 7 = grade 2, 8 or 9 = grade 3). Hormone receptor (HR) status (estrogen receptor (ER), progesterone receptor (PR)) was ascertained using immunohistochemical (IHC) testing, tumors with ≥ 10% nuclear staining were considered positive for the respective antibody. HER2 protein expression was determined by IHC using HercepTest™ (DAKO corp., CA, USA). HER2 gene amplification was determined by dual-color fluorescent in situ hybridization (FISH) using PathVysion^® ^HER2 DNA probe kit and Paraffin pretreatment kit (both Abbot-Vysis, Inc., Downers Grove, IL, USA). HER2 was considered positive when scored 3+ on the IHC staining and/or the ratio of HER2 signal to chromosome 17 signal in 60 cells by FISH analysis scored > 2.2.

### Genotyping

In sporadic breast cancer case patients, DNA was extracted from archived paraffin-embedded non-malignant breast tissues using HP PCR Template Preparation Kit (Roche Diagnostics GmbH, Mannheim, Germany). In familial breast cancer cases and controls, genomic DNA was extracted from whole blood using the FlexiGene DNA Kit 250 (Qiagen GmbH, Hilden, Germany).

Genotyping for the *KRAS *variant was carried out using the 5' nuclease assay (TaqMan; Applied Biosystems, Werterstadt, Germany). The reaction employed TaqMan Genotyping PCR Master Mix, forward primer 5-GCCAGGCTGGTCTCGAA-3, reverse primer 5-CTGAATAAATGAGTTCTGCAAAACAGGTT-3, probe-1 VIC-CTCAAGTGATTCACCCAC-MGB and probe 2 FAM-CAAGTGATGCACCCAC-MGB, as previously described [7]. Analysis was performed using the ABI PRISM^® ^7900HT Sequence Detection System and SDS 2.4 software (Applied Biosystems, Werterstadt, Germany). Some of the samples (0.7%) failed to be genotyped due to poor DNA quality. Assay reliability was assessed by random selection of 5% of samples in which genotypes were confirmed by sequencing using the 3500 Genetic Analyzer (Applied Biosystems, Werterstadt, Germany). Concordance rate was 100%.

### Statistical analysis

Descriptive and summary statistics were used to describe patient and breast tumor characteristics in the dataset. Observed genotype frequencies were tested for deviation from Hardy-Weinberg equilibrium with the chi-square goodness-of-fit test. The homozygous wild-type genotype, as determined by the more common of the homozygous genotypes, served as a reference category, with the heterozygous genotype and homozygous variant genotypes being collapsed into one category. Associations between the *KRAS *genotype and breast tumor characteristics in relation to HRT use were assessed using chi-square test. Odds ratios (ORs) for breast cancer risk and the corresponding 95% confidence intervals (CI) were calculated using logistic regression analysis. All reported p values are two-sided and considered statistically significant if p < 0.05. Analyses were performed using SPSS 19.0 software package (SPSS, Chicago, IL, USA).

## Results

The study population consisted of 530 postmenopausal sporadic breast cancer cases, 165 familial breast cancer cases (including N = 20, who test positive for *BRCA1 *mutations and N = 9, who test positive for *BRCA2 *mutations) and 270 postmenopausal control women with no history of breast and/or ovarian cancer. The mean age for sporadic breast cancer cases, familial breast cancer cases and controls was 60.45 ± 5.84, 39.75 ± 11.52 and 60.10 ± 5.85 years, respectively.

Genotype frequencies were close to those expected under Hardy-Weinberg equilibrium in both cases and controls (p > 0.05). No evidence of association was observed between the *KRAS *variant and risk of sporadic and familial breast cancer - either among *BRCA *carriers or non-*BRCA *carriers. The *KRAS *variant allele was detected in 17.2% of sporadic breast cancer cases and in 18.2% of all familial cases (10.3% of *BRCA *carriers - 10.0% of *BRCA1 *and 11.1% of *BRCA2 *carriers and in 19.9% of non-*BRCA *carriers). These frequencies were not statistically significantly different from the prevalence of the variant allele in controls (17.8%, Table 1).

**Table 1:** *KRAS *genotype frequencies in the study population

Study population	Genotype	Frequency N %	OR (95% CI)	p value
Controls	wild (TT)	221/269 (82.2)	1.0	
	variable (TG/GG)	48/269 (17.8)		

Sporadic cases	wild (TT)	434/524 (82.8)	0.96 (0.65-1.40)	0.814
	variable (TG/GG)	90/524 (17.2)		

Familial cases	wild (TT)	135/165 (81.8)	1.02 (0.62-1.69)	0.929
	variable (TG/GG)	30/165 (18.2)		

*BRCA *carriers	wild (TT)	26/29 (89.7)	0.53 (0.15-1.83)	0.316
	variable (TG/GG)	3/29 (10.3)		

*BRCA1 *carriers	wild (TT)	18/20 (90.0)	0.51 (0.12-2.28)	0.379
	variable (TG/GG)	2/20 (10.0)		

*BRCA2 *carriers	wild (TT)	8/9 (88.9)	0.58 (0.07-4.71)	0.606
	variable (TG/GG)	1/9 (11.1)		

Non-*BRCA *carriers	wild (TT)	109/136 (80.1)	1.14 (0.68-1.93)	0.623
	variable (TG/GG)	27/136 (19.9)		

We further evaluated whether the *KRAS *variant associates with a particular breast tumor characteristic. Analyses were carried out for all sporadic breast cancer cases, sporadic breast cancer cases using HRT (≥ 1 year of HRT use) and sporadic breast cancer cases not using HRT (0 < 1 years of HRT use).

Approximately one third of the sporadic breast cancer cases (n = 157, 29.6%) were using HRT prior to diagnosis, 14.3% for short-term (1 < 5 years of HRT use) and 15.3% for long-term (≥ 5 years of HRT use). Among HRT users, more than two thirds (n = 131, 71.2%) were using combined estrogen plus progestin HRT (Table 2).

**Table 2:** Distribution of HRT use among sporadic breast cancer cases

Patients characteristics		Sporadic breast cancer cases N (%)
HRT use		
	Nonusers: 0 < 1 years	373 (70.4)
	Short-term users: 1 < 5 years	76 (14.3)
	Long-term users: ≥ 5 years	81 (15.3)
	Missing	0
Regimen of HRT*		
	Estrogen only	53 (28.8)
	Estrogen plus progestin	131 (71.2)
	Missing	2

* Among those who ever used HRT

The vast majority of patients (n = 444, 84.9%) had invasive ductal carcinoma, followed by invasive lobular carcinoma (n = 62, 11.9%) and other special types of carcinoma (n = 17, 3.2%) of which there were mucinous, tubular, cribriform and medullary carcinomas. Distributions of other breast tumor characteristics are presented in Table 3.

**Table 3:** Distribution of breast tumor characteristics among sporadic breast cancer cases

Tumor characteristics		Sporadic breast cancer cases N (%)
Histologic type		
	Ductal	444 (84.9)
	Lobular	62 (11.9)
	Special-type	17 (3.2)
	Missing	7
Tumor size		
	≤ 20 mm	349 (67.2)
	21-50 mm	152 (29.3)
	> 50 mm	18 (3.5)
	Missing	11
Histopathologic grade		
	1	89 (17.1)
	2	227 (43.6)
	3	205 (39.3)
	Missing	9
Tubular formation		
	1	33 (6.3)
	2	143 (27.5)
	3	344 (66.2)
	Missing	10
Nuclear atypia		
	1	16(3.1)
	2	267(51.3)
	3	237(45.6)
	Missing	10
Mitotic index		
	1	219(42.3)
	2	141(27.2)
	3	158(30.5)
	Missing	12
Vascular invasion		
	No	403(79.3)
	Yes	105(20.7)
	Missing	22
Lymph node involvement		
	No	288(57.5)
	Yes	213(42.5)
	Missing	29
HR status		
	ER-PR-	83(15.7)
	ER + and/or PR+	444(84.3)
	Missing	3
HER2 status		
	HER2-	449(87.7)
	HER2+	63(12.3)
	Missing	18

The prevalence of the *KRAS *variant was evenly distributed between HR^+ ^and HR^- ^tumors (Table 4). The *KRAS *variant was statistically significantly more often associated with HER2^+ ^(42.9%) than HER2^- ^(13.3%) tumors and with tumors of higher histopathologic grade - score 3 (28.6%) vs. score 1 and 2 (9.6%). However, both associations were detected only in HRT users (Tables 4 and 5).

**Table 4:** *KRAS *genotype frequencies and HR status and HER2 status according to HRT stratification

Study population	Genotype	Frequency		p	Frequency p		p
		N(%)		value	N(%)		value
					
		HR^+^	HR^-^		HER2^+^	HER2^-^	
All	wild(TT)	363(82.9)	69(83.1)	0.955	49(77.8)	372(84.0)	0.218

	variable(TG/GG)	75(17.1)	14(16.9)		14(22.2)	71(16.0)	

Nonusers: HRT < 1 year	wild(TT) variable(TG/GG)	250(81.7) 56(18.3)	52(85.2) 9(14.8)	0.508	41(83.7) 8(16.3)	255(82.8) 53(17.2)	0.879

Users: HRT 1 years	wild(TT)	113(85.6)	17(77.3)	0.318	**8(57.1)**	**117(86.7)**	0.004

	variable(TG/GG)	19(14.4)	5(22.7)		**6(42.9)**	**18(13.3)**	

Statistically significant results are shown **in bold**

**Table 5:** *KRAS *genotype frequencies and histopathologic grade according to HRT stratification

Study population	Genotype	Frequency		p value
		N(%)		
			
		1/2	3	
All	wild(TT)	261(84.2)	165(80.5)	0.276
	variable(TG/GG)	49(15.8)	40(19.5)	
Nonusers: HRT < 1 year	wild(TT)	167(81.1)	130(83.3)	0.578
	variable(TG/GG)	39(18.9)	26(16.7)	

Users: HRT 1 years	wild(TT)	**94(90.4)**	**35(71.4)**	0.003
	variable(TG/GG)	**10(9.6)**	**14 (28.6)**	

Statistically significant results are shown **in bold**

To determine which of the three parameters (tubular formation, nuclear atypia, mitotic index) contributed to the higher histopathologic grade among HRT users carrying the variant allele, we evaluated the association of the *KRAS *variant with the particular parameter of the histopathologic grade among HRT users. *KRAS *variant was statistically significantly more often associated with tumors of marked nuclear atypia - score 3 (24.2%) vs. score 1 and 2 (8.9%) and with tumors of higher mitotic index - score 3 (28.9%) vs. score 1 and 2 (9.7%) (Table 6). On the other hand, the prevalence of the *KRAS *variant was not statistically significantly different in regards to what percent of the tumor formed normal duct structures (Table 6).

**Table 6:** *KRAS *genotype frequencies and histopathologic grade components among HRT users

Histopathologic grade components	Genotype	Frequency		p value
		N(%)		
			
		1/2	3	
Tubular formation	wild(TT)	54(87.1)	75(83.3)	0.525
	variable(TG/GG)	8(12.9)	15(16.7)	
Nuclearatypia	wild(TT)	**82(91.1)**	**47(75.8)**	0.010
	variable(TG/GG)	**8(8.9)**	**15(24.2)**	

Mitoticindex	wild(TT)	**102(90.3)**	**27(71.1)**	0.004
	variable(TG/GG)	**11(9.7)**	**11(28.9)**	

Statistically significant results are shown **in bold**

No statistically significant difference among *KRAS *variant carriers and non-carriers was noted in the distribution of histologic type of the tumor, tumor size, vascular invasion and lymph node involvement (data not shown).

## Discussion

The present study provides no evidence of association between the *KRAS *variant and risk of sporadic and of familial breast cancer - either among *BRCA *mutation carriers or non-*BRCA *mutation carriers.

The lack of association between the *KRAS *variant and sporadic postmenopausal breast cancer is in line with the previous findings reported by Paranjape and colleagues [11]. Although the *KRAS *variant was significantly associated with triple negative breast cancer in premenopausal women, this association was not observed for postmenopausal women regardless of HR and HER2 status [11].

In contrast to Hollestelle and colleagues [10], who found increased frequency of the *KRAS *variant among cases from *BRCA1 *positive families, we did not observe an association between the *KRAS *variant and either cases from *BRCA1*, *BRCA2 *or non-*BRCA *breast cancer families. The reason for this discrepancy might be insufficient power due to our medium-sized familial breast cancer population, however, relatively narrow confidence intervals suggest that the effect of a larger sample would not be substantial. Furthermore, expansion of the number of *BRCA1 *mutation carriers by including other family members in addition to the index cases in the study by Hollestelle and colleagues did also not improve significance, nor did the *KRAS *variant appear to modify breast cancer risk for *BRCA1 *mutation carriers [10]. Since *BRCA1 *mutations have been consistently associated with increased risk of triple negative breast cancer, Paranjape and colleagues evaluated whether the observed association of the *KRAS *variant with premenopausal triple negative breast cancer was only due to its association with carriers of *BRCA1 *mutation. They found no association between the *KRAS *variant and *BRCA1 *mutations, however, the *KRAS *variant was associated with a *BRCA1 *mutation-like gene expression signature [11]. This implies that there might be an increased oncogenic risk in the presence of the *KRAS *variant, but other mechanisms that uniquely down-regulate BRCA1 activity are assumed to be involved [11,14]. Both studies [10,11], including our own, are based on small-sized *BRCA1 *positive populations, therefore, validation in a larger cohort of *BRCA1 *mutation carriers is warranted.

Since the association of the *KRAS *variant with triple negative breast cancer risk reported by Paranjape and colleagues was noted only for premenopausal women, we carried out further analyses investigating the association between the *KRAS *variant and breast tumor characteristics in sporadic postmenopausal breast cancer cases stratified by HRT use. This article shows for the first time that the *KRAS *variant is more often associated with HER2^+ ^tumors and tumors of higher histopathologic grade - the *KRAS *variant was enriched in tumors of marked nuclear atypia and of higher mitotic index. Intriguingly, aforementioned associations were detected only in HRT users. These findings support the notion that there might be a meaningful interaction between the *KRAS *variant and hormonal exposure.

We previously showed that tumors arising in women taking HRT have a more favorable prognostic profile. Tumors in HRT users were significantly smaller with lower grade and lower mitotic index compared to tumors in nonusers [15]. The present analysis, on the other hand, revealed that HRT use in women carrying the *KRAS *variant allele is associated with HER2 overexpressed and poorly-differentiated breast tumors, both indicators of a worse prognosis. The plausable mechanism for this switch caused by the *KRAS *genotype might be the cross-talk between steroid hormone-, growth factor- and let-7 miRNA-directed pathways. It has been postulated that aberrations in growth factor pathways could dramatically influence steroid hormone action [16]. The *KRAS *oncogene is an early player in many growth factor signal transduction pathways and its overexpression can lead to increased activation of the RAF/MEK/mitogen activated protein kinase (MAPK) pathway, which in turn phosphorylate and thereby activate ERα [17]. Although there was not a substantial change in *KRAS *mRNA, Paranjape and colleagues reported an enrichment of both *NRAS *mutant and MAPK activation signatures in breast tumors that had the *KRAS *variant [11]. Another explanation could involve reduced expression of let-7 miRNA family members in *KRAS *variant-associated breast tumors [6,11]. Indeed, recently identified compelling evidence demonstrated that let-7 miRNAs target ERα and thereby repress estrogen signaling by causing a halt in cell proliferation and apoptosis [18]. Taken together, these observations suggest that due to the *KRAS *variant-driven up-regulation of growth factor-directed pathways and down-regulation of let-7 miRNAs, ERα and its downstream events might be up-regulated. Therefore, it is conceivable that ERα mediated cellular growth might be even more prominent in the case of additional exogenous estrogen intake. This was confirmed by our results showing the *KRAS *variant to be more often associated with tumors of marked nuclear atypia and of higher mitotic index in HRT users. An independent validation in another cohort is still required before any firm conclusions can be drawn.

## Conclusion

Given the increasing literature on a critical role of the *KRAS *variant in human cancers, more studies are needed to unravel the functional role of the *KRAS *variant and delineate the exact mechanism of the putative cross-talk between the *KRAS *variant and steroid hormone exposure. Modification of the effect of HRT use on breast cancer risk by the *KRAS *variant shown in our study is highly reasonable. Further insight into this is important, as it may eventually result in the ability to identify postmenopausal women who are particularly susceptible to breast cancer when exposed to surplus exogenous hormones for longer periods.

## Abbreviations

CI: Confidence interval; EOC: Epithelial ovarian cancer; E: Estrogen; ER: Estrogen receptor; ERE: Estrogen response element; FISH: Fluorescent in situ hybridization; HBOC: Hereditary breast and ovarian cancer; HER2: Human epidermal growth factor receptor 2; HPF: High-power fields; HR: Hormone receptor; HRT: Hormone replacement therapy; IHC: Immunohistochemistry; MAPK: Mitogen activated protein kinase; NSCLC: Non-small-cell lung cancer; OR: Odds ratio; PR: Progesterone receptor; SNP: Single nucleotide polymorphism.

## Competing interests

The authors declare that they have no competing interests.

## Authors' contributions

JZC, VS, KG and SN designed the investigation. JZC carried out the molecular genetic studies, analyzed the data, interpreted the results and drafted the manuscript. SN edited the manuscript. All authors read and approved the final manuscript.

## Pre-publication history

The pre-publication history for this paper can be accessed here:

http://www.biomedcentral.com/1471-2407/12/105/prepub

## References

[B1] Bartel DP MicroRNAs: genomics, biogenesis, mechanism, and function Cell 2004 116 281 297 10.1016/S0092-8674(04)00045-5 14744438

[B2] Esquela-Kerscher A Slack FJ Oncomirs - microRNAs with a role in cancer Nat Rev Cancer 2006 6 259 269 10.1038/nrc1840 16557279

[B3] Kent OA Mendell JT A small piece in the cancer puzzle: microRNAs as tumor suppressors and oncogenes Oncogene 2006 25 6188 6196 10.1038/sj.onc.1209913 17028598

[B4] Johnson SM Grosshans H Shingara J Byrom M Jarvis R Cheng A *RAS *is regulated by the let-7 microRNA family Cell 2005 120 635 647 10.1016/j.cell.2005.01.014 15766527

[B5] Chin LJ Ratner E Leng S Zhai R Nallur S Babar I A SNP in a let-7 microRNA complementary site in the *KRAS *3' UTR increases non-small cell cancer risk Cancer Res 2008 68 8535 8540 10.1158/0008-5472.CAN-08-2129 18922928 PMC2672193

[B6] Takamizawa J Konishi H Yanagisawa K Tomida S Osada H Endoh H Reduced expression of the let-7 microRNAs in human lung cancers in association with shortened postoperative survival Cancer Res 2004 64 3753 3756 10.1158/0008-5472.CAN-04-0637 15172979

[B7] Christensen BC Moyer BJ Avissar M Ouellet LG Plaza SL McClean MD A let-7 microRNA-binding site polymorphism in the *KRAS *3' UTR is associated with reduced survival in oral cancers Carcinogenesis 2009 30 1003 1007 10.1093/carcin/bgp099 19380522 PMC2691138

[B8] Ratner E Lu L Boeke M Barnett R Nallur S Chin LJ A *KRAS*-variant in ovarian cancer acts as a genetic marker of cancer risk Cancer Res 2010 70 6509 6515 10.1158/0008-5472.CAN-10-0689 20647319 PMC2923587

[B9] Pharoah PDP Palmieri RT Ramus SJ Gayther SA Andrulis IL Anton-Culver H The role of *KRAS *rs61764370 in invasive epithelial ovarian cancer: implications for clinical testing Clin Cancer Res 2011 17 3742 3750 10.1158/1078-0432.CCR-10-3405 21385923 PMC3107901

[B10] Hollestelle A Pelletier C Hooning M Crepin E Schutte M Look M Prevalence of the variant allele rs61764370 T > G in the 3' UTR of *KRAS *among Dutch *BRCA1*, *BRCA2 *and non-*BRCA1/BRCA2 *breast cancer families Breast Cancer Res Treat 2011 128 79 84 10.1007/s10549-010-1080-z 20676756 PMC3735357

[B11] Paranjape T Heneghan H Lindner R Keane FK Hoffman A Hollestelle A A 3'- untranslated region *KRAS *variant and triple-negative breast cancer: a case-control and genetic analysis Lancet Oncol 2011 12 377 386 10.1016/S1470-2045(11)70044-4 21435948 PMC3488438

[B12] Cerne JZ Gersak K Novakovic S The influence of the genetic variant within miRNA-binding site in estrogen receptor alpha gene on the risk of breast cancer in postmenopausal women on hormone replacement therapy Cancer Biomark 2011 8 123 128 22012767 10.3233/DMA-2011-0813 PMC13015870

[B13] Elston CW Ellis IO Pathological prognostic factors in breast cancer. I. The value of histological grade in breast cancer: experience from a large study with long-term follow-up Histopathology 1991 19 403 410 10.1111/j.1365-2559.1991.tb00229.x 1757079

[B14] Kumar MS Swanton C KRAS 3'-UTR variants and stratification of breast-cancer risk Lancet Oncol 2011 12 318 319 10.1016/S1470-2045(11)70065-1 21435949

[B15] Cerne JZ Frkovic-Grazio S Gersak K Breast tumor characteristics in hormone replacement therapy users Pathol Oncol Res 2011 17 917 923 10.1007/s12253-011-9403-x 21678110

[B16] Nicholson RI McClelland RA Robertson JFR Gee JMW Involvement of steroid hormone and growth factor cross-talk in endocrine response in breast cancer Endocr Relat Cancer 1999 6 373 387 10.1677/erc.0.0060373 10516852

[B17] Santen RJ Song RX McPherson R Kumar R Adam L Jeng MH The role of mitogen-activated protein (MAP) kinase in breast cancer J Steroid Biochem Mol Biol 2002 80 239 256 10.1016/S0960-0760(01)00189-3 11897507

[B18] Zhao Y Deng C Wang J Xiao J Gatalica Z Recker RR Let-7 family miRNAs regulate estrogen receptor alpha signaling in estrogen receptor positive breast cancer Breast Cancer Res Treat 2011 127 69 80 10.1007/s10549-010-0972-2 20535543

